# Methadone-Mediated Autonomic Functioning of Male Patients with Heroin Dependence: The Influence of Borderline Personality Pattern

**DOI:** 10.1371/journal.pone.0037464

**Published:** 2012-05-22

**Authors:** Wei-Lieh Huang, Yu-Hsuan Lin, Terry B. J. Kuo, Li-Ren Chang, Ying-Zai Chen, Cheryl C. H. Yang

**Affiliations:** 1 Department of Psychiatry, National Taiwan University Hospital, Yun-Lin Branch, Yunlin, Taiwan; 2 Department of Psychiatry, National Taiwan University Hospital, Taipei, Taiwan; 3 Institute of Brain Science, National Yang-Ming University, Taipei, Taiwan; 4 Sleep Research Center, National Yang-Ming University, Taipei, Taiwan; 5 Research Center for Adaptive Data Analysis, National Central University, Taoyuan, Taiwan; University of Western Brittany, France

## Abstract

**Background:**

We hypothesize that the population with borderline personality shows different autonomic response to methadone compared to individuals with other personalities. This study applies heart rate variability (HRV) measurements and the Tridimensional Personality Questionnaire (TPQ) to examine this hypothesis.

**Methodology/Principal Findings:**

Forty-four male patients with heroin dependence were recruited from a methadone maintenance treatment program. Eight personality patterns were classified according to the TPQ norm used in Taiwan. The borderline pattern (BP, composed of high novelty seeking, high harm avoidance and low reward dependence) and the other personality patterns (OP) were separated into two groups. We compared the HRV profiles between the BP and OP groups. Correlation and regression analysis were performed to clarify relationship between HRV differences and the borderline index (BI, a new concept defined by us, which is calculated as novelty seeking + harm avoidance – reward dependence). The HRV targets investigated included low frequency (LF) power, high frequency (HF) power, total power (TP), normalized LF (LF%), and LF/HF. No baseline HRV parameters showed any inter-group difference. The BP group had a significantly lower ΔHF and a higher ΔLF/HF than the OP group. The personality dimension, reward dependence, showed a negative correlation with ΔLF/HF and ΔLF%. BI was negatively correlated with ΔHF and positively correlated with ΔLF/HF and ΔLF%.

**Conclusions/Significance:**

Borderline personality individuals show increased sympathetic activity and decreased parasympathetic activity compared to other personalities after taking methadone. The results support the hypothesis that there is an interaction between borderline personality and autonomic modulation.

## Introduction

Heroin dependence is a complex psychiatric disorder that causes worldwide social and economic problems [1]. It has a high relapsing rate and therefore harm reduction becomes an important treatment option [2]. Methadone is a widely used agent for maintenance therapy and has been proved to reduce illicit opioid use and criminality [3]. Many factors affect the outcome of heroin abusers who undertake methadone maintenance therapy (MMT) [4]. Personality is a frequently mentioned one [5], [6]. Research has revealed that the personality features of patients with heroin dependence involve anxiety, impulsiveness, and social detachment [7]. Many of them fulfill the criteria for borderline personality disorder (BPD) as defined in Diagnostic and Statistical Manual of Mental Disorders, Fourth Edition (DSM-IV) [5], [8]. The clinical features of BPD include fear of abandonment, explosive and self-injurious behaviors, and emotional dysregulation [9]. The BPD population group shows a low retention rate and high illicit substance exposure when undergoing MMT [5], [10], [11]. However, these studies have focused on psychological explanations for the poor outcome of MMT and the biological mechanism of substance use behavior among BPD individuals remains a mystery.

Disturbance of emotional regulation is a core feature of BPD [12], [13]. Polyvagal theory, constructed by Porges, provides a connection between autonomic functioning and emotional dysregulation [14], [15], [16]. The theory distinguishes between two branches of vagus nerve. The older branch, originating from the dorsal motor nucleus, is associated with primitive threat responses, such as freezing. The newer branch, connected to the nucleus ambiguous, is responsible for the modulation of social affiliation behaviors and is only found in mammals. A threatening situation causes inhibitory signals from the newer vagal branch to decrease rapidly, and results in a sympathetically dominant flight-or-fight response. The phenomenon is called vagal withdrawal. An abnormal pattern of vagal withdrawal may explain the severe emotional disturbance in BPD individuals [17]. Based on this theory, Weinberg et al. found BPD subjects to have increased sympathetic activity and decreased parasympathetic activity compared to controls when involved with a social stressor task [18]. We wonder if a similar response may also exist when facing a physiological stimulus, such as treatment with methadone or other substances. Such a concept could provide new insights into the irregular compliance found among BPD individuals.

Heart rate variability (HRV) has been broadly used to measure autonomic nervous system (ANS) functioning [19], [20]. Frequency domain analysis of HRV differentiates between high frequency (HF) power, low frequency (LF) power, and very-low frequency (VLF) power [21]. HF is believed to be an index of vagal modulation, whereas normalized LF and the LF/HF ratio reflect sympathetic function [20], [21]. Our previous study discloses that opiates have influence on HRV. HF in heroin abusers is lower than that in healthy population. It implies that long-term exposure of opiates may reduce vagal modulation. On the other hand, heroin abusers show increased HF after taking methadone, which might originate from the relief of opiate withdrawal symptoms [22]. Researches exploring personality dimensions and HRV have revealed that the anxiety trait is related to HF [23], [24], [25]. Among tools evaluating personality dimensions, the Tridimensional Personality Questionnaire (TPQ) is frequently used in studies of BPD [26], [27], [28]. Borderline personality is related to a pattern of high novelty seeking (NS), high harm avoidance (HA), and low reward dependence (RD) [29], [30]. TPQ allows researchers to analyze borderline personality from a measurable and quantitative viewpoint distinct from DSM-IV. We consider that combining TPQ and HRV should give further insights into borderline personality and the biological response to methadone. In addition, since features of borderline personality can be understood in terms of its composition, namely NS, HA and RD, we propose that a combination of the three scores is able to reflect the degree of borderline personality. In the presented study, we define borderline pattern according to TPQ distribution rather than criteria of DSM-IV. It is because that some subjects revealing borderline personality “trait” do not necessarily fulfill definition of borderline personality “disorder”. However, the trait may still show specific presentations in ANS.

There are two major purposes explored in the presented study. One is to examine if the borderline pattern and other personality patterns have different ANS responses to methadone. The other is to clarify if the ANS response can be predicted by a quantitative value of personality. The two goals are based on following hypotheses. Firstly, we assume that methadone will have a less vagal elevating or less sympathetic reducing effect in the borderline group than in the other personality groups. Secondly, we hypothesize that a quantitative index that is composed of high NS, high HA and low RD is relatable to measurable ANS functions.

## Methods and Materials

### Participants and Procedures

We performed this study in Department of Psychiatry, National Taiwan University Hospital (NTUH), Yun-Lin Branch, in Taiwan. The Institutional Review Board of NTUH approved the study program. In order to investigate the personality and HRV among heroin abuser, we recruited adults from the MMT program at the hospital. The participants were all male. This was for two reasons. Firstly, most patients in our MMT program are male. Secondly, HRV is affected by the menstruation cycle, which is a factor that is hard to control for [31]. The participants’ ages ranged from 25 to 55 years old, which was chosen so the information gathered was that of a formed and stable personality. Patients with mental retardation, current psychotic disorder, diabetes mellitus, hypertension, and cardiovascular diseases, were excluded. After completing the informed consent, a subject spent 1.5 hour answering the questionnaires and receiving two episodes of HRV measurement. The first HRV measurement was carried out before the individual took their regular methadone dose and the second measurement was taken after drinking the methadone. The interval between the two measurements was 60 minutes. All data on each subject was gathered in a single morning (9am–12am) in order to avoid any influence from the individual’s circadian rhythm. In demographic data section, the various factors known to influence HRV, such as age, smoking, baseline blood pressure, body mass index (BMI), and exercise level, were recorded.

### Measurements

Four validated questionnaires were used in the presented study. The Tridimensional Personality Questionnaire (TPQ) was chosen to evaluate personality traits, which is our major analysis target. Beck Depression Inventory (BDI), the Subjective Opiate Withdrawal Scale (SOWS) and the Opiate Treatment Index (OTI) were also administered in order to clarify the influence of psychological state and substance use behavior on HRV.

TPQ is a self-administered questionnaire with 100 true-false questions [30]. It is designed to measure biological component of personality. The Chinese version used in our study was developed by Chen et al [32]. There are three major dimensions in TPQ and these are novelty seeking (NS), harm avoidance (HA), and reward dependence (RD). NS consists of NS1 (exploratory excitability), NS2 (impulsivity), NS3 (extravagance) and NS4 (disorderliness). HA consists of HA1 (anticipatory worry), HA2 (fear of uncertainty), HA3 (shyness with strangers) and HA4 (fatigability and asthenia). RD consists of RD1 (sentimentality), RD3 (attachment) and RD4 (dependence). RD2 (persistence) has been proved to be an independent dimension relative to other RD concepts and is not collected into the total RD score. According to the normative data for TPQ in Taiwan, we divided our sample to eight personality patterns (for example, the norm of NS/HA/RD in Taiwan is 13.2/13.8/13.5, a profile with a NS/HA/RD of 18/17/10 is coded as ++-) [32]. Cloninger’s temperament cube theory assumes that each pattern is associated with a personality category [30], [33]. In the presented study, the borderline pattern (BP) and other patterns (OP) were separated into two groups in order to examine our hypothesis. In addition, we believe that some borderline features, such as affective instability and impulsive behavior may be explained by combining the three major dimensions. To quantitatively calculate the borderline features into a single value, we develop the concept of a “borderline index” (BI). BI is calculated from NS total + HA total – RD total  =  BI score, because high NS, high HA, low RD is the main feature of borderline personality in Cloninger’s temperament cube theory [30].The dimensional patterns have been found to be correlated with scores of categorical assessing tools, such as the Personality Disorder Questionnaire Revised [33].

BDI is a self-report inventory with 21 multiple choice questions and measures both the cognitive and vegetative symptoms of depression [34]. SOWS is a self-rating scale the aim of which is to measure the severity of opioid withdrawal syndrome [35]. It contains 16 symptoms, which the intensity of which is rated on a 5-point scale. OTI is a validated tool for evaluating the substance use behaviors of patients with opioid dependence [36], [37], [38]. In the presented study, we focus on the influence of the estimated consumption amount (Q score) of various substances. The prescribed benzodiazepine was calculated into tranquilizer Q score. Each Q score is calculated by the formula: Q  =  (q_1_+ q_2_)/(t_1_+ t_2_), where q_1_, q_2_ are the substance amount used on the last and the second last use occasion, t_1_ means the interval between the last day and next to last day of substance exposure and t_2_ is the interval between the second and third last day of substance exposure. The current methadone dose was also recorded. This comes from the individual’s medical records and is reliable.

### Heart Rate Variability

We used a five-minute measurement of HRV in the present study. Subjects were asked to sit in a relaxed manner but not to fall asleep. A HRV analyzer (SS1C, Enjoy Research Inc., Taiwan) was adopted for signal acquisition, storage and processing. An 8-bit analog-to-digital converter with a sampling rate of 512 Hz was used to record the signals. The digitized ECG signals were analyzed online and simultaneously stored on a hard disk for offline verification. The computer algorithm then identified each QRS complex and rejected ventricular premature complex or noise based on likelihood using a standard QRS template. We resampled and interpolated normal and stationary R-R interval values at a rate of 7.11 Hz to produce continuity in the time domain. 2048 data points over 288 seconds was produced by the interpolation and was used for the subsequent fast Fourier transformation. After deleting the baseline shift, a Hamming window was used to attenuate the leakage effect. Our algorithm estimated the power spectrum density for each time segment. The power spectrum was subsequently quantified into the standard frequency-domain measurements, including high frequency (HF) power (between 0.15 and 0.4 Hz), low frequency (LF) power (between 0.04 and 0.15 Hz), very low frequency (VLF) power (between 0.003 and 0.04 Hz), total power (TP), normalized LF (LF%), and LF/HF.

### Statistical Analysis

The demographic data, psychological state, substance use behaviors and the TPQ profiling were compared between BP and OP groups by independent t test. The differential effects of the personality patterns (BP and OP) and methadone (before and after taking methadone) were assessed by an independent t test and a paired t test. The relationships between HRV, TPQ, and other factors known to affect HRV were analyzed using correlation and regression analysis.

## Results

Demographic, psychological state, and substance use behavior data for all subjects, are shown in Table 1. The mean age is 36.98±7.01 years old. All participants had been educated more than 9 years. 29.55% of them were married. There were 22 subjects in the BP group and 22 in the OP group. Their demographic data, BMI, and blood pressure, did not show any significant difference. There is also no inter-group difference in BDI score, SOWS score, and Q score for any recorded substance. The average methadone dose of BP and OP are 38.50±25.47 mg/day and 37.82±15.23 mg/day, which are similar to other studies performed in Taiwan [3]. No significant inter-group difference in SOWS score and methadone dose suggests that the status of tolerance and withdrawal were similar in BP and OP.

**Table 1:** Demographic, psychological state and substance use behaviors compared between the borderline pattern and other patterns.

	Total(N = 44)	Borderline pattern (N = 22)	Other patterns (N = 22)			
	Mean (±SD)/Number (%)	Mean (±SD)/Number (%)	Mean (±SD)/Number (%)	F	χ2	*p* value
**Demographics**						
**Age (year)**	36.98 (±7.01)	36.68 (±7.89)	37.27 (±6.17%)	1.150		0.783
**Gender (male)**	44 (100.00%)	22 (100.00%)	22 (100.00%)		NA	NA
**Marital status (married)**	13 (29.55%)	8 (36.36%)	5 (22.73%)		0.983	0.322
**Educational level (>9 years)**	27 (61.36%)	12 (54.55%)	15 (68.18%)		0.863	0.353
**Exercise level (hour/day)**	0.33 (±0.95)	0.18 (±0.37)	0.47 (±1.26)	2.258		0.413
**BMI (kg/m^2^)**	23.38 (±3.44)	23.68 (±3.63)	23.08 (±3.30)	0.004		0.568
**SBP (mmHg)**	124.09 (±13.15)	122.91 (±12.18)	125.33 (±14.29)	0.309		0.552
**DBP (mmHg)**	77.14 (±11.26)	77.45 (±10.37)	76.81 (±12.38)	0.125		0.854
**Psychological state**						
**BDI score**	11.52 (±11.24)	10.05 (±10.32)	13.00 (±12.15)	0.644		0.390
**SOWS score**	7.07 (±5.51)	7.18 (±5.57)	6.95 (±5.58)	0.086		0.893
**Substance use behaviors**						
**Current methadone dose (mg/day)**	38.16 (±20.75)	38.50 (±25.47)	37.82 (±15.23)	3.149		0.915
**Heroin Q score**	0.62 (±1.12)	0.50 (±0.70)	0.74 (±1.43)	1.749		0.482
**Alcohol Q score**	0.82 (±3.43)	0.13 (±0.29)	1.51 (±4.80)	7.477		0.194
**Cigarette Q score**	17.64 (±9.47)	15.91 (±9.33)	19.36 (±9.51)	0.229		0.231
**Polysubstance Q score**	18.88 (±11.01)	16.73 (±9.66)	21.02 (±12.04)	0.095		0.200
**Other opioid Q score**	0.18 (±0.82)	0.32 (±1.13)	0.05 (±0.21)	5.535		0.280
**Amphetamine Q score**	0.03 (±0.15)	0.02 (±0.05)	0.05 (±0.21)	1.835		0.548
**Tranquilizer Q score**	0.09 (±0.34)	0.13 (±0.43)	0.05 (±0.21)	1.742		0.452
**Hallucinogen Q score**	0.11 (±0.75)	0.23 (±1.07)	0.00 (±0.00)	4.410		0.329

* *p*<0.05,

** *p*<0.001.

BMI, body mass index; SBP, systolic blood pressure; DBP, diastolic blood pressure; BDI, Beck Depression Inventory; SOWS, Subjective Opiate Withdrawal Scale; Q score, estimated consumption amount score.

Considering all subjects, the mean NS total score, mean HA total score and mean RD total score are 15.05±3.34, 16.33±2.80 and 11.66±2.08, respectively. Comparing BP and OP, the three major dimensions NS, HA, RD all reveal a significant inter-group difference. Among the subdimensions, only NS2 (impulsivity) is significantly different between the two groups. As we expected, the borderline index is significantly higher for the BP group compared to the OP (Table 2).

**Table 2:** TPQ data between borderline pattern and other pattern individuals.

	Total (N = 44)	Borderline pattern (N = 22)	Other patterns (N = 22)			
	Mean (±SD)	Mean (±SD)	Mean (±SD)	F	χ2	*p* value
**NS1 (exploratory excitability)**	3.56 (±1.45)	3.89 (±1.35)	3.23 (±1.51)	0.409		0.132
**NS2 (impulsivity)**	3.08 (±1.40)	3.75 (±1.39)	2.41 (±1.06)	1.062		0.001*
**NS3 (extravagance)**	3.43 (±1.58)	3.50 (±1.47)	3.36 (±1.71)	0.257		0.780
**NS4 (disorderliness)**	4.98 (±1.58)	5.25 (±1.46)	4.70 (±1.68)	0.329		0.258
**HA1 (anticipatory worry)**	3.79 (±1.47)	4.09 (±1.34)	3.50 (±1.56)	0.076		0.186
**HA2 (fear of uncertainty)**	3.64 (±1.33)	3.86 (±1.16)	3.41 (±1.47)	0.941		0.262
**HA3 (shyness with strangers)**	3.27 (±1.32)	3.50 (±1.14)	3.05 (±1.46)	0.004		0.257
**HA4 (fatigability and asthenia)**	5.64 (±1.42)	5.77 (±1.41)	5.50 (±1.44)	0.036		0.530
**RD1 (sentimentality)**	3.91 (±1.26)	3.59 (±1.53)	4.23 (±0.81)	4.189		0.095
**RD2 (persistence)**	4.77 (±1.32)	4.80 (±1.49)	4.75 (±1.17)	0.606		0.910
**RD3 (attachment)**	5.93 (±1.27)	5.64 (±1.14)	6.23 (±1.34)	0.058		0.123
**RD4 (dependence)**	1.82 (±1.57)	1.64 (±1.46)	2.00 (±1.69)	1.482		0.449
**NS total**	15.05 (±3.34)	16.39 (±1.86)	13.71 (±3.95)	6.944		0.007*
**HA total**	16.33 (±2.80)	17.22 (±2.25)	15.44 (±3.06)	2.446		0.034*
**RD total**	11.66 (±2.08)	10.86 (±1.52)	12.45 (±2.29)	3.470		0.009*
**Borderline index**	19.72 (±5.17)	22.74 (±4.08)	16.70 (±4.36)	0.003		<0.001**

* *p*<0.05,

** *p*<0.001.

RD total does not include RD2.

Borderline index  =  NS total + HA total – RD total.

TPQ, Tridimensional Personality Questionnaire; NS, novelty seeking; HA, harm avoidance; RD, reward dependence.

**Figure 1:**
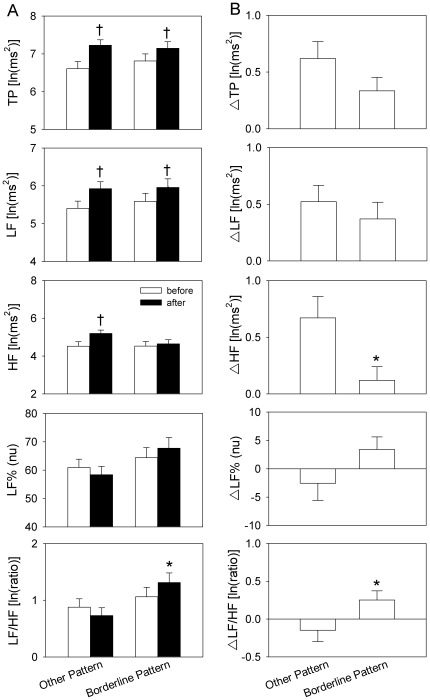
Comparison of heart rate variability (HRV) parameters before and after taking methadone. (A) After methadone consumption, low frequency (LF) power and total power (TP) in the borderline pattern (BP) group and in the other patterns (OP) group are both elevated, while high frequency (HF) power only shows a significant elevation in OP group. Noticeably, the normalized LF (LF%) and LF/HF increase slightly after taking methadone among the BP group. (B) The HF and LF/HF are significantly different between the BP and OP groups. *p<0.05 *vs*. OP, †*p*<0.05 *vs*. before.

Among LF, HF, TP, LF% and LF/HF, before taking methadone, there was no significant inter-group difference. Among the HRV parameters after taking methadone, only LF/HF is significantly higher in BP compared to the OP group (Fig. 1A). In addition, comparing the HRV profiles before and after taking methadone by paired t test, BP and OP reveal divergent manifestations. Methadone caused a significant elevation in LF and TP among both groups. However, with HF, only the OP group show a significantly increase after taking methadone (Fig. 1A). Noticeably, the methadone effect with respect to LF% and LF/HF is an elevation among the BP group and a reduction among the OP, although no significance difference was revealed by paired t test. When we examine the difference in HRV profiles, which are defined as values after methadone subtracted from the values before methadone, there are some positive findings. ΔHF is significantly lower among the BP group, while ΔLF/HF is higher among the BP group (Fig. 1B).

**Figure 2:**
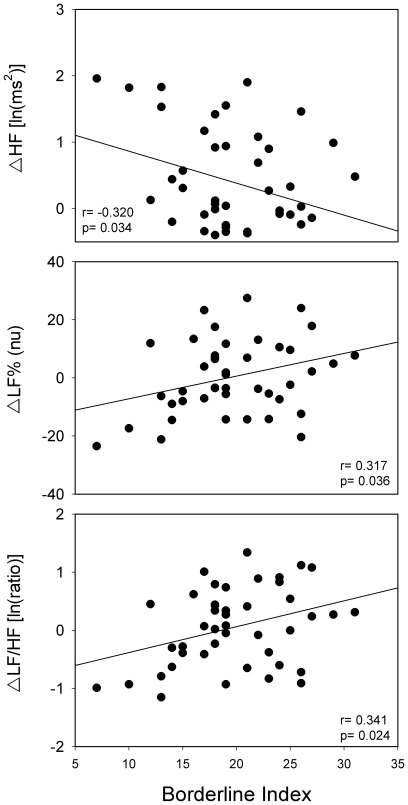
A simple linear regression model of the borderline index and differences in high frequency power of heart rate variability (HF), normalized low frequency power of heart rate variability (LF%), ratio of LF/HF (LF/HF) before and after taking methadone. The borderline index, defined by novelty seeking score + harm avoidance score – reward dependence score  =  BI score from the Tridimensional Personality Questionnaire is used as a proposed value for evaluating the severity of borderline features.

The relationship between TPQ profile and HRV parameters was further examined by correlation analysis. Considering the traditional three TPQ major dimensions, RD is negatively correlated with ΔLF% (r = −0.368, *p* = 0.014) and ΔLF/HF (r = −0.354, *p* = 0.018), while NS and HA do not show any significant correlation with HRV differences. The combinative value BI reveals a more meaningful connection with HRV. ΔHF is negatively correlated with BI, while ΔLF% and ΔLF/HF show positive correlations with BI. Multiple linear regression analysis reveals that BI is predictive of ΔHF, ΔLF%, and ΔLF/HF. Age, BMI, SOWS score, and BDI score have no significant meaning with respect to the regression model (Table 3). Figure 2 is a simple linear regression model of BI and the relationships with ΔHF, ΔLF%, ΔLF/HF.

**Table 3:** Predictors of HRV difference after controlling for age, BMI, exercise level, SOWS score, BDI score, current methadone dose and substance Q scores by multiple linear regression analysis.

	△HF		△LF%		△LF/HF	
R^2^	0.147		0.324		0.441	
	β	*p* value	β	*p* value	β	*p* value
**Age**	0.004	0.983	−0.088	0.590	−0.029	0.854
**BMI**	0.078	0.674	−0.108	0.522	−0.089	0.595
**Exercise level**	−0.095	−0.595	0.333	0.045*	0.200	0.200
**SOWS score**	0.063	0.725	0.094	0.567	0.083	0.586
**BDI score**	0.118	0.512	−0.171	0.295	−0.108	0.492
**Current methadone dose**	0.004	0.983	−0.243	0.168	−0.337	0.039*
**Heroin Q score**	0.040	0.833	−0.246	0.157	−0.233	0.174
**Alcohol Q score**	0.287	0.111	−0.281	0.086	−0.331	0.037*
**Cigarette Q score**	−0.108	0.546	−0.002	0.991	0.096	0.560
**Tranquilizer Q score**	−0.104	0.563	−0.044	0.786	−0.094	0.538
**Borderline index**	−0.383	0.037*	0.465	0.007*	0.448	0.009*

* *p*<0.05,

** *p*<0.001.

HRV, heart rate variability; BMI, body mass index; SOWS, Subjective Opiate Withdrawal Scale; BDI, Beck Depression Inventory; Q score, estimated consumption amount score; HF, high frequency power of HRV, LF, low frequency power of HRV, LF%, normalized low frequency power of HRV.

## Discussion

This is the first study to demonstrate diverging ANS reactions after methadone consumption in borderline and other personalities. In summary, there are two major findings in the presented study. Firstly, after taking methadone, ΔHF and ΔLF/HF are significantly different between the BP and OP groups. BP shows a lower ΔHF but a higher ΔLF/HF, which corresponds with our first hypothesis. Secondly, the proposed severity index of borderline personality, BI, is negatively correlated with ΔHF and positively correlated with ΔLF% and ΔLF/HF, which supports our second hypothesis.

The OP groups shows a significant elevation in HF and a slightly reduction of LF% and LF/HF after drinking methadone. However, after methadone consumption, the BP group shows limited HF elevation together with a LF and LF/HF increase. This implies that members of the BP group tend to have higher sympathetic and lower parasympathetic activities after taking methadone. Other factors known to affect HRV, such as age, BMI, exercise level, emotional state, and substance exposure, do not show any inter-group differences. Similarly, the SOWS score and the heroin Q scores for the two groups indicate that the differences in HRV response are not due to noncompliance or opioid withdrawal. Multiple linear regression analysis also confirmed that personality profiles have a predictive meaning in terms of HRV response. This strongly suggests that there is a relationship between personality pattern and HRV response. Our results are similar to Weinberg et al.’s study and are compatible with the polyvagal theory [17], [18]. Furthermore, BPD individuals have been found to have a higher risk developing stroke and ischemic heart disease, which can also be understood in terms of sympathovagal imbalance [39].

The clinical features of BPD can be explained from a ANS perspective. When suffering an unpleasant or stressful event, the BPD population lacks self-stabilizing ability. The elevation in sympathetic tone overwhelms their deficient vagal modulation, which induces an intense flight-or-fight response [18]. This results in affective instability, difficulty controlling anger, destructive behavior, and even self-damaging. The limited increase in HF after drinking methadone implies that central nervous system depressants have only a partial inhibiting or relaxing effect on members of the BPD population. The phenomenon of positive partial reinforcement results in a trend whereby BPD patients take more drugs to stabilize themselves, leading to addictive behaviors [40]. The above mechanisms also generate noncooperation, a low retention rate, and a poor prognosis for MMT among these individuals.

Cloninger hypothesized that NS, HA and RD are related to different brain systems. NS is associated with dopamine, while HA is related to serotonin in dorsal raphe nucleus and gamma-amino butyric acid; RD is bound up with norepinephrine [41], [42]. The high correlation between HRV parameters and the borderline index, implies the complexity of the borderline personality features. If we calculate three dimensions separately, only the correlation between RD and ΔLF% and ΔLF/HF are significant. Norepinephrine is the most important neurotransmitter both in the dimension RD and sympathetic nervous system [31], [42]. HA total score, which reflects the degree of anxiety trait, did not show significant correlation with ΔHF, ΔLF%, or ΔLF/HF. The relation between HRV and monoaminergic biology in BPD subjects warrants further investigation.

One limitation of this study is the definition of the borderline group. Although a positive correlation exists, the borderline pattern of TPQ is not identical to the BPD defined in DSM-IV. Personality disorders in DSM-IV are not mutually-exclusive and as a result several types of personality disorder can be diagnosed in one patient. This is a different approach to the TPQ pattern classification. Therefore, whether the results of TPQ pattern can be applied into clinical BPD needs more examinations. Furthermore, we only recruited male subjects into this study, but 75% of patients with a BPD diagnosis are female [9]. However, a combination of high novelty seeking and high harm avoidance has been reported to be more frequent in male BPD patients [43]. In addition, in terms of limitations, the subjects who completed all questionnaires and HRV measurements are likely to be the more cooperative individuals. Therefore, our sample cannot reflect all patients with heroin dependence. The results also need a larger sample size to confirm.

Our study gives a reasonable connection between borderline personality, methadone, and autonomic function. The result can be extended to explain the emotional dysregulation and addictive behaviors of BPD individuals. As a pilot study in this field, our results provide a new insight into the ANS modulation of patients with heroin dependence. Integration with BPD as defined in the DSM-IV and an investigation of female subjects seem likely to be the follow-up goals. A comprehensive design is needed to validate the findings described above and to clarify the mechanisms involved.
